# Microstructural Variations in the Bone of *Pygoscelis antarctica* (Aves, Sphenisciformes) During the Postnatal Ontogeny

**DOI:** 10.3390/biology15090703

**Published:** 2026-04-30

**Authors:** Luis Marcial Garat, Marianella Talevi, Carolina Acosta Hospitaleche

**Affiliations:** 1 Instituto de Investigación en Paleobiología y Geología (IIPG), Universidad Nacional de Río Negro (UNRN), Av. Roca 1242, General Roca R8332EXZ, Río Negro, Argentina; mtalevi@unrn.edu.ar; 2 Consejo Nacional de Investigaciones Científicas y Tecnológicas (CONICET), Godoy Cruz 2290, Buenos Aires C1425FQB, Buenos Aires, Argentina; 3 División Paleontología Vertebrados, Museo de La Plata, Facultad de Ciencias Naturales y Museo (FCNyM), Universidad Nacional de La Plata (UNLP), Paseo del Bosque S/N, La Plata B1900FWA, Buenos Aires, Argentina

**Keywords:** osteohistology, bone compaction, chicks, juvenile, penguin, Neornithes

## Abstract

Ontogenetic changes in bone microstructure provide key information on growth patterns and functional differentiation in birds. This study describes microanatomical and histological variation in the appendicular and axial skeleton across an ontogenetic series of *Pygoscelis antarctica*. Fifty-two thin sections were analyzed, representing five chicks of different ages, a juvenile, and an adult. Early stages are characterized by thin, highly vascularized cortices and extensive trabecular bone surrounding a large medullary cavity. The chick sequence shows progressive periosteal deposition, trabecular expansion, and increasing osteosclerosis. In the juvenile, secondary compaction and the initial development of the inner circumferential layer appear together with the onset of extensive remodeling. The adult specimen exhibits fully developed outer and inner circumferential layers, lamellar cortical tissue, and marked secondary remodeling, with differential medullary reduction among skeletal elements. Compared with *Aptenodytes patagonicus*, *P. antarctica* shows earlier and more pronounced medullary reduction and secondary trabecular compaction in the forelimbs, likely related to its shorter dependency period and earlier initiation of marine locomotion. In contrast, hindlimbs retain a medullary cavity and follow sex-linked pathways of medullary modification: males develop an inner circumferential layer, whereas females show trabecular bone followed by secondary compaction.

## Simple Summary

We analyzed how the bone microstructure of the Antarctic penguin *Pygoscelis antarctica* changes during growth. To explore this, 52 thin sections from the appendicular and axial skeleton of several individuals representing different life stages were analyzed. Our results show that, in the youngest chicks, bones have thin walls, are more vascularized, and have abundant trabecular tissue surrounding a large internal cavity. As the chicks grow, bone tissue gradually thickens, trabeculae expand, and the skeleton becomes progressively denser. In the juvenile, the bones begin to show stronger internal organization, including the first signs of inner circumferential layers and more intense bone remodeling. Adult bones display fully developed structural layers, dense lamellar tissue, and extensive secondary remodeling, although the size of the internal cavity varies among skeletal elements. Comparisons with previous results for the larger king penguin *Aptenodytes patagonicus* show that *P. antarctica* exhibits an earlier reduction in the internal cavity and greater compaction in the forelimbs. These differences likely reflect its shorter period of parental care and the earlier onset of swimming behavior.

## 1. Introduction

Osteohistological analysis is a valuable tool for understanding the microanatomical and microstructural changes that occur during ontogeny. In Sphenisciformes, bone growth dynamics may be influenced by ecological and ethological factors (e.g., [1,2,3,4]). Locomotion, duration of parental dependence, and the onset of chick independence vary considerably among penguin species [5], particularly between species with prolonged reproductive cycles and delayed chick independence, such as *Aptenodytes patagonicus*, and those with accelerated development and early independence, such as *Pygoscelis antarctica* [6,7].

Although Meister [8] and Margerie et al. [9] analyzed the microanatomy and histology of chicks and adults of *A. patagonicus*, the first detailed osteohistological study based on an ontogenetic series in Sphenisciformes was conducted by Canoville [10] and, more recently, by Canoville et al. [11]. These authors focused on 34 specimens of *A. patagonicus*, including chicks and adults. In their study, three chick stages (I, II, and III) were defined, during which variations in parental feeding were analyzed and used as a basis for the histological characterization of forelimb and hindlimb bones. Their results indicated that the hindlimbs of *A. patagonicus* matured earlier and exhibited a higher degree of bone compaction than the forelimbs. These osteohistological observations not only allowed for the identification of bone maturation patterns but also provided a framework for linking bone structure to locomotor behavior during early life stages. This suggests that varying degrees of biomechanical stimulation may influence bone remodeling and the compaction of skeletal elements [11].

In this context, the present study aims to describe and compare microanatomical and histological variation in the appendicular and axial skeleton of *Pygoscelis antarctica* throughout postnatal ontogeny. Based on the analysis of specimens representing different developmental stages (five chicks of different ages, one juvenile, and one adult), we seek to identify the primary changes associated with skeletal maturation, locomotor adaptation, and other developmental patterns characteristic of the species.

## 2. Materials and Methods

Seven specimens of *Pygoscelis antarctica* from the Ornithology Section of the Vertebrate Zoology Division, La Plata Museum (MLP-O), La Plata, Buenos Aires Province, Argentina, were used in this study. The analyzed ontogenetic series was constructed from specimens whose relative age was estimated based on size, degree of ossification, and textural aging of the skeletal elements, following the proposal of Acosta Hospitaleche and Picasso [12]. The sex of the specimens included in the ontogenetic series is mostly unknown. Accordingly, specimens were assigned to three ontogenetic stages: pre-juvenile, juvenile, and adult. The pre-juvenile stage was further subdivided into five categories (Chick I, II, III, IV, and V), based on limb size. Only Chick II was identified as a male, whereas the sex of the remaining individuals, including the juvenile and adult specimens, could not be determined. The juvenile stage was defined by active periosteal growth and incomplete ossification of the epiphyses. The adult stage was characterized by the cessation of periosteal growth and complete ossification of the epiphyses.

**Figure 1:**
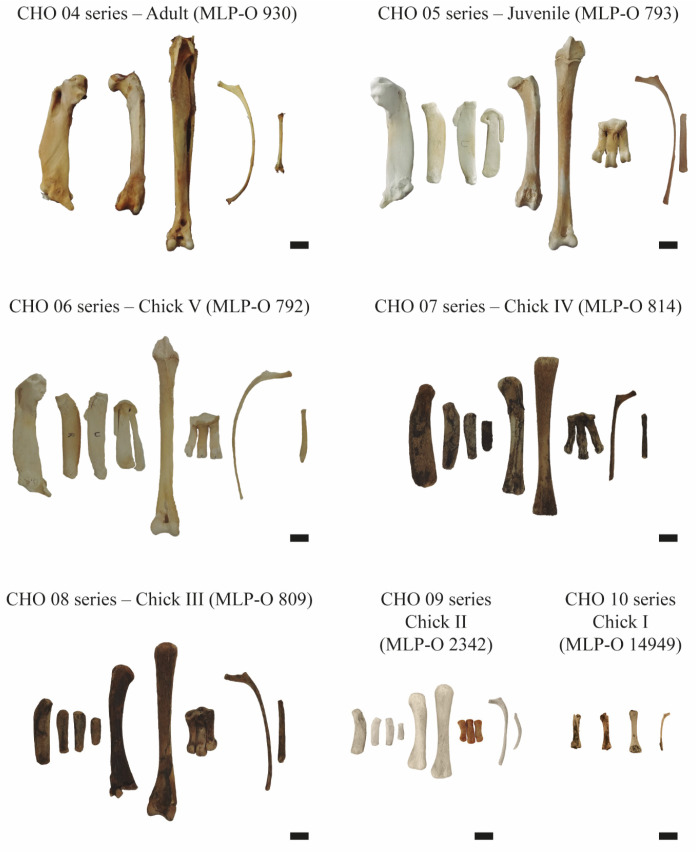
Ontogenetic series of *Pygoscelis antarctica*. From left to right; (CHO 04 series—Adult) humerus, femur, tibiotarsus, vertebral and sternal rib; (CHO 05 series—Juvenile) humerus, radius, ulna, carpometacarpus, femur, tibiotarsus, tarsometatarsus, vertebral and sternal rib; (CHO 06 series—Chick V) humerus, radius, ulna, carpometacarpus, tibiotarsus, tarsometatarsus, vertebral and sternal rib; (CHO 07 series—Chick IV) humerus, radius/ulna, radius/ulna, carpometacarpus, femur, tibiotarsus, tarsometatarsus, vertebral and sternal rib; (CHO 08 series—Chick III) humerus, radius/ulna, radius/ulna, carpometacarpus, femur, tibiotarsus, tarsometatarsus, vertebral and sternal rib; (CHO 09 series—Chick II) humerus, radius, ulna, carpometacarpus, femur, tibiotarsus, tarsometatarsus, vertebral rib and sternal; (CHO 10 series—Chick I) femur, femur, tibiotarsus and vertebral rib. Humerus, femur, tibiotarsus, and tarsometatarsus in cranial view. Radius, ulna, and carpometacarpus in ventral view. Vertebral and sternal rib in lateral view. Scale 1 cm.

Histological sections (CHO; Histological collection of the Ornithology Section) of the appendicular and axial skeleton were prepared from different specimens of *P. antarctica* (MLP-O 930, MLP-O 793, MLP-O 792, MLP-O 814, MLP-O 809, MLP-O 2342, and MLP-O 14949) (Figure 1). A total of 52 thin sections (Table 1) were prepared at the Laboratorio de Secciones Delgadas of the Instituto de Investigación en Paleobiología y Geología, General Roca, Río Negro Province, Argentina. These are deposited in the Ornithology Section of the Vertebrate Zoology Division, La Plata Museum. Thin sections were obtained at the midshaft of the bones, following the procedure described by Chinsamy and Raath [13], as this region is less affected by metaphyseal remodeling and preserves the primary cortical bone tissue, allowing for more reliable histological comparisons [14], and examined using a petrographic microscope (Zeiss Axio Imager, Carl Zeiss AG, Oberkochen, Germany) under plane- and cross-polarized light. Images were captured with a digital camera (Zeiss Axiocam 105 color, Carl Zeiss AG, Oberkochen, Germany) and processed using Adobe Photoshop 2020 v21.0 (image editing) and Adobe Illustrator 2022 v26.0 (figure preparation).

Among the pre-juvenile categories, specimen Chick II (CHO 09 series) was described in detail, as it exhibited the most representative characteristics of the pre-juvenile stage and included all analyzed skeletal elements, allowing for direct comparisons with the remaining pre-juvenile ontogenetic stages.

## 3. Results

Some bones within the same specimen share microanatomical and histological traits. Accordingly, we first describe the general characteristics of the specimen, and then outline the specific characteristics of each bone, as applicable.

It should be noted that the juvenile and adult stages are each represented by a single specimen. Therefore, the histological features described for these stages should be considered as preliminary trends rather than definitive characteristics of the species.

### 3.1. Microanatomy and Histology

#### 3.1.1. Adult Specimen

The middle section of the bone elements analyzed in the adult specimen (CHO 04 series) exhibits compact bone tissue (Supplementary Figure S1). No medullary cavity is present in the humerus; instead, a series of small intertrabecular spaces resulting from secondary compaction is observed, indicating a higher degree of bone compaction than in the hindlimbs. In contrast, the hindlimb elements (femur and tibiotarsus) and the sternal rib show a medullary cavity with an irregular margin. In the vertebral rib, the medullary region is occupied by extensive intertrabecular spaces.

**Figure 2:**
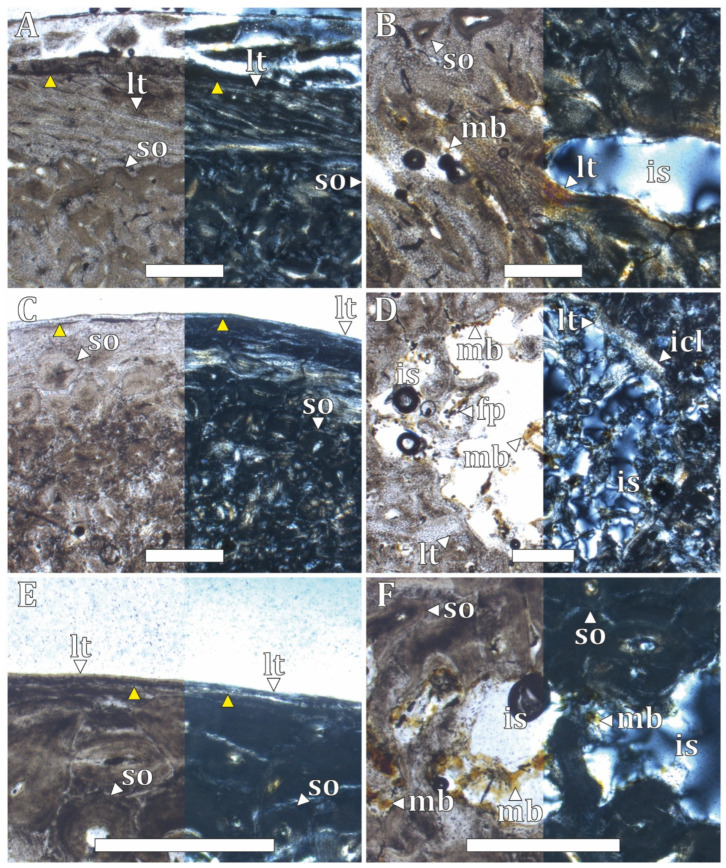
Histological features of the humerus, femur, and vertebral rib of the adult specimen of *Pygoscelis antarctica*. (**A**,**B**) humerus, (**C**,**D**) femur, and (**E**,**F**) vertebral rib, under normal transmitted light (**left**) and under polarized light (**right**). (**A**,**C**,**E**) cortical and perimedullary region. (**B**,**D**,**F**) medullary region. (fp) framboidal pyrite, (icl) inner circumferential layer, (is) intertrabecular space, (lt) lamellar tissue, (mb) medullary bone, (so) secondary osteon, and (yellow triangle) LAG. Scale 500 μm.

In the cortical region of the adult specimen (CHO 04 series) (Figure 2A,C,E), the tissue consists of a lamellar bone matrix. In the humerus, this tissue displays a laminar vascular pattern (Figure 2A). By contrast, in the femur, tibiotarsus, and both vertebral and sternal ribs, the cortical tissue is avascular and exhibits a line of arrested growth (LAG) extending along its entire circumference (Figure 2C,E).

In the perimedullary region (Figure 2A,C,E), several secondary osteons are observed remodeling a primary woven-fibered bone matrix. These osteons are distinguished by clear cement lines and are distributed mainly in a longitudinal vascular pattern. In the tibiotarsus, a nutrient canal partially lined by lamellar bone is identified in the perimedullary region.

The medullary region shows the greatest variability among the analyzed skeletal elements (Figure 2B,D,F). In the humerus, the medullary region is composed of secondarily compacted trabecular bone with small intertrabecular spaces (Figure 2B). In the femur and tibiotarsus, a medullary cavity with an irregular margin is present and connects with adjacent intertrabecular spaces (Figure 2D). Additionally, in the femur, a partially remodeled inner circumferential layer (ICL) surrounds the medullary cavity (Figure 2D). In contrast, no medullary cavity is observed in the vertebral rib, which instead shows a network of intertrabecular spaces invaded by bone trabeculae (Figure 2F). The sternal rib, however, exhibits a well-defined medullary cavity without the presence of an ICL.

In all sections, medullary bone surrounds the medullary spaces (Figure 2B,D,F), and vascularization is predominantly longitudinal. Osteocyte lacunae associated with dynamic osteogenesis (DO) are smaller, discoidal, and aligned with the orientation of the bone fibers, whereas osteocyte lacunae formed by static osteogenesis (SO) are larger, circular, and occur within remnants of the primary bone matrix. No Sharpey’s fibers were observed.

#### 3.1.2. Juvenile Specimen

In the juvenile specimen (CHO 05 series), the midshaft regions of the analyzed bone elements show compact bone tissue (Supplementary Figure S1). In the forelimb (humerus, radius, ulna, and carpometacarpus), several intertrabecular spaces are present within the medullary region. The radius, ulna, and carpometacarpus retain a small medullary cavity, whereas the humerus lacks a distinct medullary cavity. The femur and tibiotarsus exhibit a medullary cavity with an irregular margin, surrounded by adjacent intertrabecular spaces that may converge with it. Additionally, bone trabecula partially invades this space. In the tarsometatarsus and the vertebral and sternal ribs, no medullary cavity is present; instead, the medullary region is occupied by irregularly shaped or circular intertrabecular spaces.

**Figure 3:**
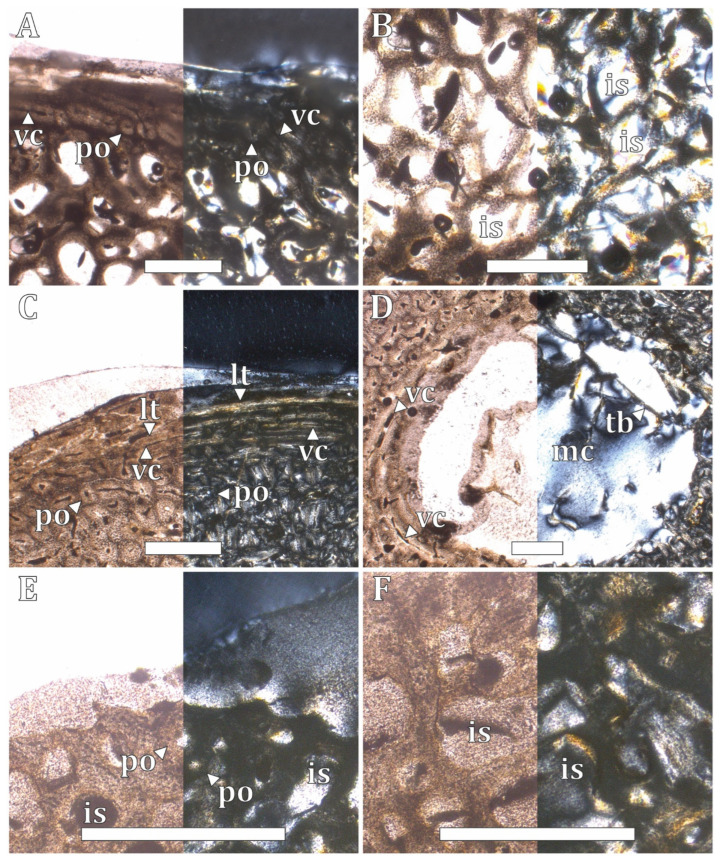
Histological features of the humerus, femur, and vertebral rib of the juvenile specimen of *Pygoscelis antarctica*. (**A**,**B**) humerus, (**C**,**D**) femur, and (**E**,**F**) vertebral rib, under normal transmitted light (**left**) and under polarized light (**right**). (**A**,**C**,**E**) cortical and perimedullary region. (**B**,**D**,**F**) medullary region. (is) intertrabecular space, (lt) lamellar tissue, (mc) medullary cavity, (po) primary osteon, (tb) trabecular bone, and (vc) vascular canal. Scale 500 μm.

In the cortical region of the juvenile specimen (CHO 05) (Figure 3A,C,E), vascularized tissue composed of a woven-fibered bone matrix with vascular channels opening towards the cortical margin is observed. In the humerus and femur (Figure 3A,C), the cortical tissue exhibits a laminar vascular pattern, whereas in the radius, ulna, and carpometacarpus, only longitudinal vascular channels are present.

In the perimedullary and medullary regions (Figure 3A–F), trabecular bone with extensive intertrabecular spaces, irregular or circular in shape, is observed. Longitudinally oriented osteons are present, and in most sections, bone remodeling is limited, with a relative abundance of primary osteons. Likewise, no evidence of remodeling is detected in the radius, carpometacarpus, tarsometatarsus, vertebral, and sternal ribs. By contrast, a nutrient canal partially lined by lamellar bone is identified on the perimedullary margins of the femur and tibiotarsus. The morphologies of the SO and DO osteocyte lacunae are well-defined, although greater development of SO osteocyte lacunae is observed in the sections. No Sharpey’s fibers or medullary tissue are present.

#### 3.1.3. Chick Specimen

In chicks, the middle section of the analyzed bone elements consists predominantly of trabecular bone, with extensive intertrabecular spaces occupying the medullary region (Supplementary Figure S1). In Chick II (CHO 09 series), except for the tarsometatarsus, a medullary cavity with an irregular margin is present and may converge with adjacent intertrabecular spaces. Differences observed among chick specimens are related to a gradual increase in cortical compaction and trabecular bone development throughout the sequence from Chick I to Chick V (Supplementary Figure S1).

**Figure 4:**
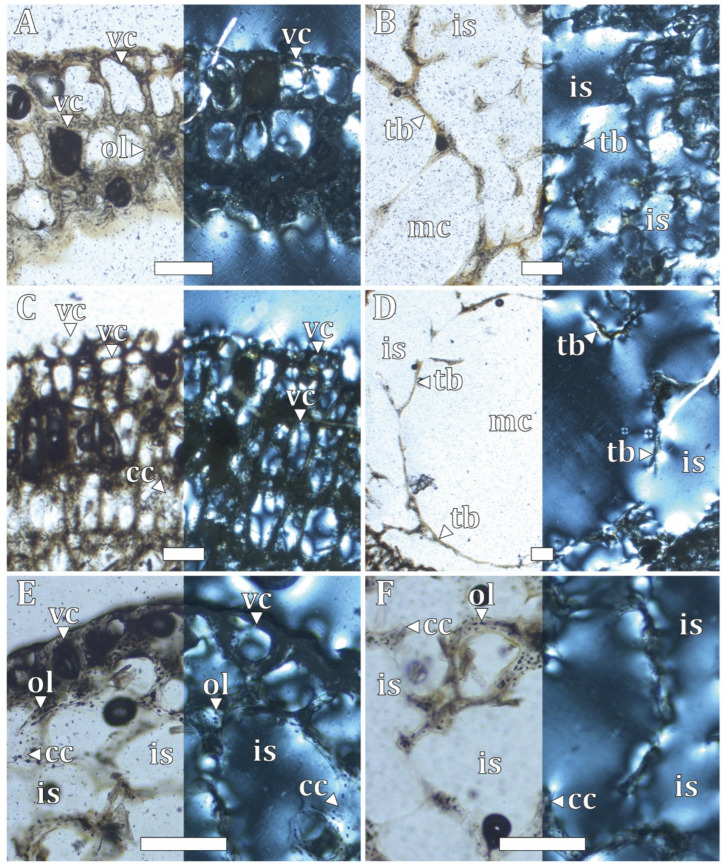
Histological features of the humerus, femur, and vertebral rib of Chick II specimen of *Pygoscelis antarctica*. (**A**,**B**) humerus, (**C**,**D**) femur, and (**E**,**F**) vertebral rib, under normal transmitted light (**left**) and under polarized light (**right**). (**A**,**C**,**E**) cortical and perimedullary region. (**B**,**D**,**F**) medullary region. (cc) calcified cartilage, (is) intertrabecular space, (mc) medullary cavity, (ol) osteocyte lacuna, (tb) trabecular bone, and (vc) vascular canal. Scale 200 μm.

In the cortical region of Chick II (CHO 09 series), a woven-fibered bone matrix with several simple vascular channels is observed, arranged longitudinally and organized in a laminar vascular pattern (Figure 4A,C,E). In addition, some vascular channels open towards the cortical margin (Figure 4A,C).

In the medullary region (Figure 4B,D,F), trabecular bone with extensive intertrabecular spaces and fine woven-fibered matrix trabeculae is present. In most sections, a medullary cavity with an irregular margin is observed. By contrast, in the tarsometatarsus, only trabecular bone composed of fine lamellae is present, with no clear histological distinction between cortical and medullary regions. In the femur, tarsometatarsus, and vertebral rib, a relatively high abundance of calcified cartilage is observed (Figure 4C,E,F).

Numerous circular osteocyte lacunae produced by static osteogenesis are present (Figure 4A). No evidence of bone remodeling, Sharpey’s fibers, or medullary bone is observed.

#### 3.1.4. Comparative Description of the Ontogenetic Series of *Pygoscelis antarctica*

**Figure 5:**
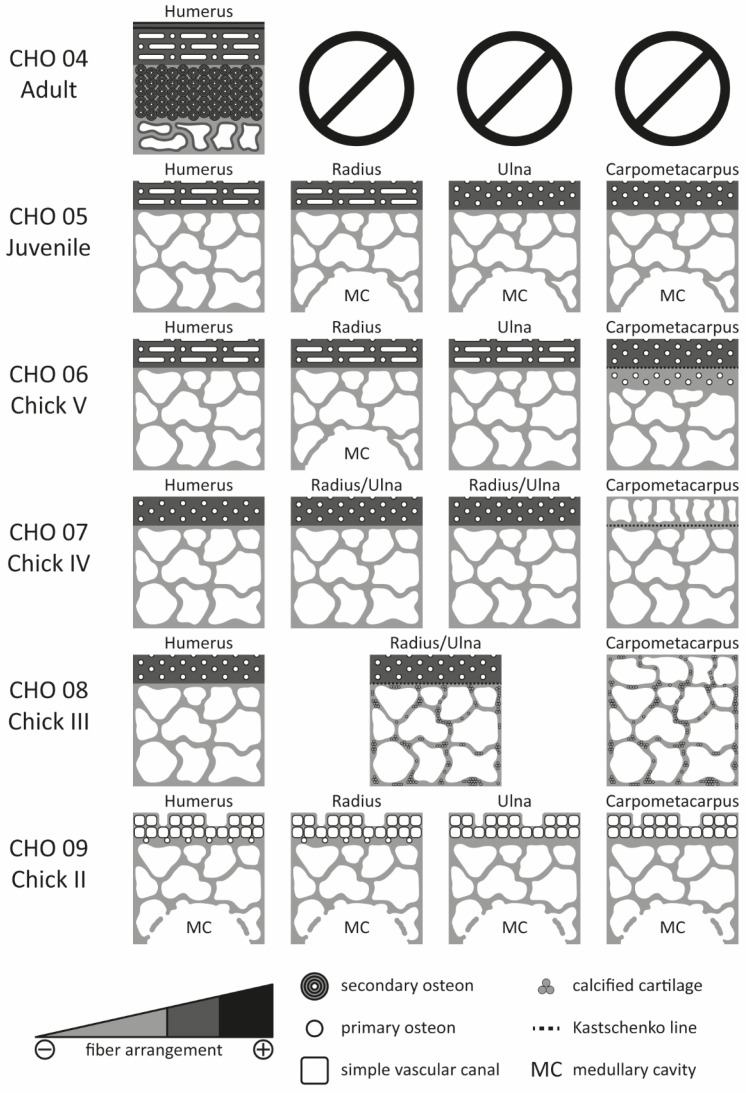
Schematic representation of the histological structures of the forelimbs in the ontogenetic series of *Pygoscelis antarctica*.

**Figure 6:**
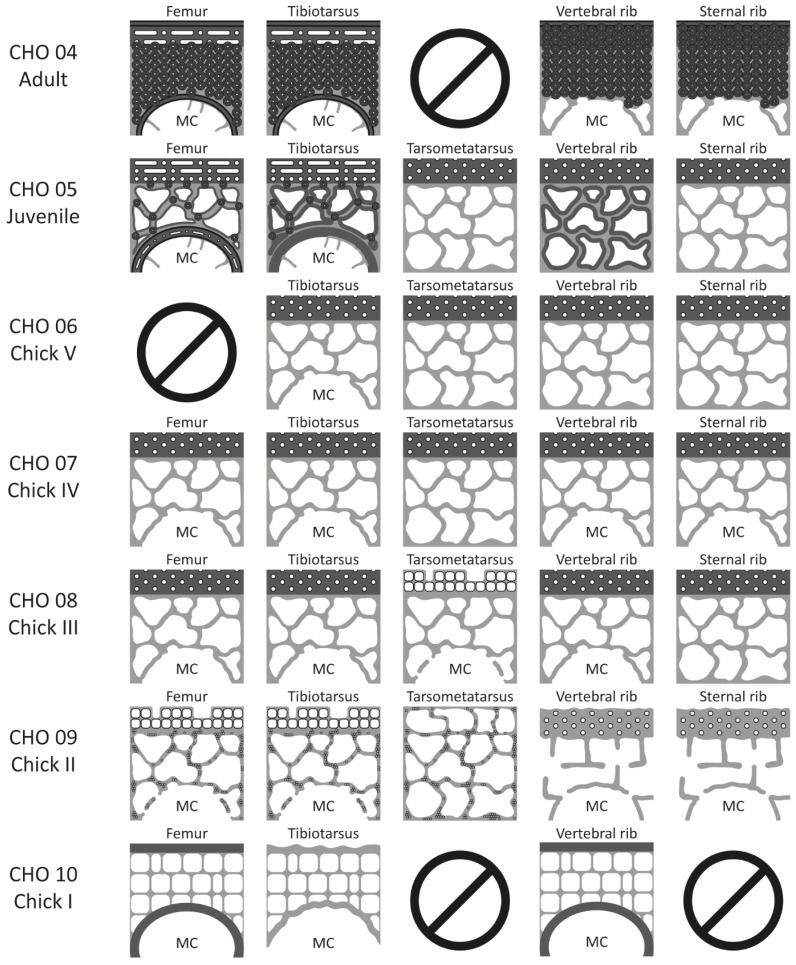
Schematic representation of the histological structures of the hindlimbs and ribs in the ontogenetic series of *Pygoscelis antarctica*. The references can be found in Figure 5.

The histological sections of *Pygoscelis antarctica* show a tendency towards osteosclerotic development throughout ontogeny in all the skeletal elements examined. Accordingly, during the early stages, a thin, compact, and highly vascularized cortical region, together with a large medullary cavity well-delimited by a layer of bone tissue, is observed (Figure 5 and Figure 6). These features reflect the high metabolic rates of the species and are consistent with biomechanical properties typically associated with flight [3,15]. This observation is particularly relevant because penguins retain this histological pattern only during their early ontogenetic phases.

Likewise, in the pre-juvenile stages (Chick I, II, III, IV, and V), intense periosteal growth is evident, characterized by a high number of vascular channels opening towards the outer margin of the cortical region and by a marked expansion of both the medullary cavity and trabecular bone. However, unlike the observations reported by Meister [8] for pre-juvenile stages, no nucleus of hyaline cartilage was preserved in the medullary region. Furthermore, vascular arrangements are exclusively longitudinal and restricted to the cortical region (Figure 5 and Figure 6).

In the juvenile stage, a marked increase in internal bone compaction is observed, occurring primarily through secondary compaction of the trabecular bone and, secondarily, through the organization and expansion of the inner circumferential layer (ICL) within the medullary region (Figure 5 and Figure 6). At this stage, a high degree of bone remodeling also becomes evident. Secondary osteons initially extend into the perimedullary and medullary regions and subsequently advance towards the cortical region.

In the adult stage, changes in both bone matrix organization and vascular patterns are observed in the cortical and medullary regions. The bone matrix is organized into a well-developed outer circumferential layer (OCL) and ICL, and vascularization tends to be predominantly laminar in these regions. In addition, secondary osteons extensively remodel the tissues of both the cortical and medullary regions (Figure 5 and Figure 6).

Regarding bone compaction, very small, irregularly shaped intertrabecular spaces lacking a preferential orientation are observed, resulting from secondary compaction of trabecular bone and producing a distinctive lattice-like appearance. It is important to distinguish this pattern of secondarily compacted trabecular bone from the reticular pattern formed by vascular channels. In the former, the small spaces represent remnants of previously larger intertrabecular spaces, whereas in the latter, the observed spaces correspond to the vascular channels themselves.

#### 3.1.5. Comparative Comments on the Forelimbs

Among all the analyzed bones, the humerus shows a clear tendency towards osteosclerotic development and a rapid reduction in the medullary cavity during the pre-juvenile stages (Figure 5) (e.g., Chick III and Chick IV). In Chick II, the humerus of *Pygoscelis antarctica* exhibits a compact but highly vascularized cortical region and trabecular bone surrounding an extensive medullary cavity. By the Chick III stage, the medullary cavity and trabecular bone expand towards the dorsal and ventral margins of the section. In Chick IV, the medullary region becomes completely segmented by bone trabeculae. During the Chick V and juvenile stages, secondary compaction of the trabecular bone begins. Subsequently, in the adult stage, the remodeling front advances over the medullary margin, replacing the secondarily compacted trabecular bone with secondary osteons. With respect to the cortical tissue, rapid growth characterizes the pre-juvenile stages (e.g., Chick II, Chick III, Chick IV, and Chick V), as indicated by numerous simple vascular channels and primary osteons embedded within a woven-fibered bone matrix. From Chick IV onwards, changes in the vascular pattern of the cortical region become evident. In earlier stages, the cortex exhibits a laminar vascular pattern with osteons organized longitudinally. However, in the Chick V, juvenile, and adult stages, this pattern becomes laminar with osteons arranged both longitudinally and circumferentially. In the adult stage, cortical development culminates in the formation of a typical OCL, composed of avascular lamellar tissue, which may or may not display lines of arrested growth (LAGs) along its extension.

Regarding the medullary spaces and vascularity of the radius, ulna, and carpometacarpus, a general trend like that observed in the humerus (namely, progressive osteosclerotic development) is recognized. However, in the midshaft regions of these elements in the juvenile stage, a rapid loss of the medullary cavity is not observed. Instead, a small medullary cavity with an irregular margin is retained, without the development of an ICL. In the absence of homologous adult elements, the complete obliteration of the medullary cavity in these bones cannot be confirmed for *P. antarctica* (Figure 5). Additionally, a Kastschenko line is observed in the radius/ulna of Chick III and in the carpometacarpus of Chick IV and Chick V, marking the first periosteal bone deposition in these growing individuals (Supplementary Figure S2). By contrast, changes in cortical vascular patterns comparable to those observed in the humerus are not detected in the carpometacarpus, which retains longitudinal vascular channels arranged in a laminar pattern throughout ontogeny.

#### 3.1.6. Comparative Comments on the Hind Limbs

The hindlimb elements analyzed also exhibit clear osteosclerotic development; however, unlike the humerus, they retain a medullary cavity in the adult stage (Figure 6). Early expansion of the medullary cavity and rapid periosteal growth are observed during the pre-juvenile stages (e.g., Chick I, II, III, and IV), as evidenced by the presence of numerous simple vascular canals and primary osteons in the cortical region. In the femur and tibiotarsus of Chick II, the cortex is compact but highly vascularized, with several simple vascular canals, and the medullary region consists of trabecular bone surrounding an extensive medullary cavity. From Chick III onwards, rapid periosteal growth and increasing compaction of the cortical region are evident. In the adult femur and tibiotarsus, bone remodeling advances markedly along the perimedullary margin, modifying the ICL of the medullary region. At this stage, cortical tissue development in the femur, tibiotarsus, and tarsometatarsus results in the formation of a typical OCL, composed of avascular lamellar tissue bearing LAGs.

With respect to medullary spaces and vascularization, the femur and tibiotarsus display several similarities but also notable differences. In the juvenile femur, the cortical tissue shows a laminar vascular organization composed of both longitudinal and circular channels. The medullary cavity is surrounded by a pseudo-lamellar matrix with circular vascular channels, with some bone trabeculae invading the medullary cavity. In the adult femur, this laminar vascular pattern of longitudinal and circular channels persists, and the tissue adjacent to the medullary cavity is organized as an ICL, again with bone trabeculae extending into the medullary space. By contrast, in the juvenile tibiotarsus, the periosteal tissue exhibits a laminar vascular organization composed exclusively of longitudinal channels, and the medullary cavity has an irregular margin, lacks an ICL, and contains invading bone trabeculae. In this element, secondary compaction of the trabecular bone is more pronounced than in the femur. In the adult tibiotarsus, however, the cortical vascular pattern becomes laminar with both longitudinal and circular channels, as observed in the adult femur, and an ICL is present, together with some bone trabeculae within the medullary cavity.

The tarsometatarsus also exhibits clear osteosclerotic development, but unlike the femur and tibiotarsus, it retains only simple vascular channels during the juvenile stage. With respect to the medullary region, a single medullary cavity is observed in Chick III and Chick IV, whereas, in subsequent stages, this cavity is replaced by several intertrabecular spaces forming part of the trabecular tissue (Supplementary Figure S3). In Chick II, the tarsometatarsus is characterized by a homogeneous tissue composed of a poorly mineralized woven-fibered bone matrix, with abundant calcified cartilage and several simple vascular channels.

#### 3.1.7. Comparative Comments on Vertebral and Sternal Ribs

The ribs of *Pygoscelis antarctica* exhibit clear osteosclerotic development and retain a small medullary cavity in the adult stage (Figure 6). In Chick I, the vertebral rib exhibits thin, highly vascularized cortical tissue and an extensive medullary cavity. A thin, avascular layer of poorly mineralized lamellar to pseudo-lamellar bone is present along both the outer and inner margins of the cortical region. In Chick II, III, and IV, expansion of the medullary cavity and progressive development of trabecular bone are observed. During the juvenile stage, secondary compaction of the trabecular bone becomes evident. In the adult stage, this tissue is largely replaced by longitudinally arranged secondary osteons because of extensive bone remodeling. This compaction is markedly more pronounced in the vertebral rib than in the sternal rib. In addition, during the pre-juvenile (Chicks I, II, III, IV, and V) and juvenile stages, numerous vascular channels, opening towards the outer margin of the cortical region, are observed. In the adult stage, remodeling fronts affect the entire cortical and medullary regions, including the OCL in the cortex. Although the sternal rib follows a broadly similar ontogenetic trajectory to that of the vertebral rib, a distinct medullary cavity cannot be identified in either element during the Chick V and juvenile stages. Instead, the medullary region is occupied by trabecular bone.

With respect to medullary spaces and vascularization, the ribs exhibit patterns comparable to those observed in the hindlimbs. However, no changes in vascular patterns are detected throughout ontogeny, as only longitudinal vascular channels are present, without a clearly developed laminar arrangement.

## 4. Discussion

### 4.1. Osteohistological Variations in the Ontogenetic Series of Pygoscelis antarctica

The ontogenetic stages established for *Pygoscelis antarctica* are not directly comparable with those defined for *Aptenodytes patagonicus* by Canoville et al. [11], as the latter are based on exact age in weeks. Because of these differences, comparisons are restricted to general microanatomical and histological characteristics rather than to specific ontogenetic stages. However, caution is warranted when interpreting the histological features observed in the juvenile and adult stages, as each ontogenetic category is represented here by a single specimen. Consequently, the traits described for these stages should be regarded as tentative and may not fully capture the intraspecific variability of *P. antarctica*.

Overall, the results obtained for the ontogenetic series of *P. antarctica* are largely similar to those described for *A. patagonicus* by Canoville et al. [11]. Shared features between the two species include: (1) a clear tendency towards osteosclerotic development; (2) rapid periosteal accretion, together with expansion of the medullary cavity and trabecular bone during the chick stages; (3) the formation of primary and secondary osteons associated with centripetal endosteal deposition; and (4) centrifugal deposition of compact bone during the juvenile and adult stages, accompanied by changes in bone matrix organization and vascular patterning.

Despite these similarities, important microstructural differences are evident between the two species. Canoville et al. [11] reported that in *A. patagonicus*, bone compaction occurred earlier in the hindlimbs (femur and tibiotarsus) than in the forelimbs (humerus and radius). In contrast, in *P. antarctica*, the humerus shows an early reduction and eventual disappearance of the medullary cavity, along with pronounced secondary compaction of trabecular bone during the pre-juvenile stages (Chicks I–V), relative to the femur. This differential compaction is particularly evident in the humerus and femur of the adult *P. antarctica* (CHO 04 series). Similar compaction patterns are observed in the radius, ulna, and carpometacarpus; however, the absence of these elements in the adult specimen of *P. antarctica* precludes confirming complete medullary cavity reduction throughout ontogeny. Nevertheless, in the forelimbs of two adult specimens of *Pygoscelis adeliae* [16], no medullary cavity was identified; it was replaced instead by markedly reduced intertrabecular spaces resulting from bone compaction. Given the close phylogenetic relationship between these species, both of which belong to the genus *Pygoscelis*, it is plausible that *P. antarctica* also lacks a medullary cavity in the radius, ulna, and carpometacarpus in adulthood.

Canoville et al. [11] attributed the earlier maturation and greater bone compaction of the hindlimbs in *A. patagonicus*, relative to the forelimbs, to the differential use of musculoskeletal elements involved in terrestrial locomotion ([17] and references therein), interpreted as an adaptation to the bipedal and terrestrial lifestyle of chicks. In addition, *A. patagonicus* is characterized by one of the longest breeding and moulting cycles among penguins [5], lasting approximately 17 months. During this period, chicks undergo prolonged fasting over winter and may take 9–12 months to attain independence [6]. By contrast, *P. antarctica* has a shorter reproductive cycle (approximately one year), and chick independence is reached after only 4–7 weeks [7]. This marked contrast in the timing of chick maturation between *A. patagonicus* and *P. antarctica* may account for the observed differences in the sequence of bone compaction between hindlimbs and forelimbs in the two species. In *P. antarctica*, early independence results in the rapid onset of foraging excursions, and consequently, the forelimbs (directly involved in swimming) begin to be used for marine locomotion within a few months of hatching. This activity likely provides sufficient biomechanical stimulation to accelerate bone compaction in the humerus, and probably throughout the forelimb, relative to the femur and tibiotarsus. In *A. patagonicus*, the delayed onset of marine locomotion may result in reduced mechanical stimulation of the forelimbs. In contrast, the greater reliance on terrestrial locomotion during early life promotes earlier bone compaction in hindlimbs. These patterns underscore the strong influence of lifestyle and activity regimes on skeletal development among penguin species.

Another notable histological difference between *A. patagonicus* and *P. antarctica* concerns the nature and timing of lines of arrested growth (LAGs). Although both species experience fasting during their annual cycle, it is considerably longer in *A. patagonicus*, lasting up to five months in some chicks before the parental feeding renews and independence occurs [18]. Castanet [19] first documented the presence of LAGs in *A. patagonicus*, based on observations of the femur of a juvenile specimen, and these growth marks have subsequently been interpreted as a result of extended periods of nutritional stress [11,19]. In contrast, fasting in *P. antarctica* typically lasts only around three weeks and occurs during the moulting period. Although *P. antarctica* does not display LAGs in the perimedullary region during juvenile stages, LAGs may be present in adult individuals, occurring within the cortical region as part of the OCL and are interpreted as indicators of somatic maturity. Similarly, Canoville et al. [11] identified an OCL with associated LAGs in adult specimens of *A. patagonicus*. Despite differences in fasting duration, body mass loss during fasting is broadly comparable between the two species, amounting to approximately 40–50% in *A. patagonicus* and around 40% in *P. antarctica* ([5,17,18,20,21,22,23], and references therein).

Finally, comparison of the ontogenetic series examined here with the observations of Meister [8] on a chick and an adult of *A. patagonicus* reveals both similarities and minor differences. In the earliest stages of *P. antarctica* (Chicks I and II), no cartilaginous core or mesenchymal cells were identified, probably as a result of post-mortem preservation conditions. Nevertheless, a relatively high abundance of calcified cartilage was observed within the primary bone tissue. In the adult *A. patagonicus* described by Meister [8], histological patterns closely resembling those observed in *P. antarctica* were documented, including a concentric organization into three regions (external, middle, and internal), high bone density, and comparable vascular arrangements.

### 4.2. Sex-Related Bone Compaction Dynamics of the Hindlimbs

The sex of the specimens analyzed is largely unknown, with the exception of Chick II. The sex-related bone compaction patterns discussed below are based on comparisons with previous studies on closely related species, particularly *Pygoscelis*. In this context, although differences in the degree of limb bone compaction related to lifestyle have been documented (e.g., Canoville et al. [11]), recent studies [16,24] have also identified microstructural differences in the type of bone compaction associated with sex, particularly in the hindlimbs of *Pygoscelis adeliae*. In this species, females exhibit a lower degree of bone compaction, a greater development of intertrabecular spaces, and the presence of medullary bone. In contrast, males show higher bone compaction, absence of medullary bone, and development of an ICL [16]. In addition, the hindlimb skeletal elements of females (femur, tibiotarsus, and tarsometatarsus), as well as the ribs (vertebral and sternal), display medullary spaces arranged in a latticework pattern (e.g., [16], Figure 2), similar to those described by Ksepka et al. [25] in the femur of *Spheniscus* sp. and in the femur and tibiotarsus of *Aptenodytes forsteri* (e.g., Ksepka et al. [25], Figure 3 and Figure 4). However, Garat et al. [16] noted that the forelimb skeletal elements of both male and female *P. adeliae* do not exhibit an ICL. Subsequent work [24] confirmed the presence of an ICL only in the hindlimbs of females; however, this layer is remodeled and ultimately lost, due to osteoclastic activity associated with medullary bone formation and calcium mobilization during eggshell production.

**Figure 7:**
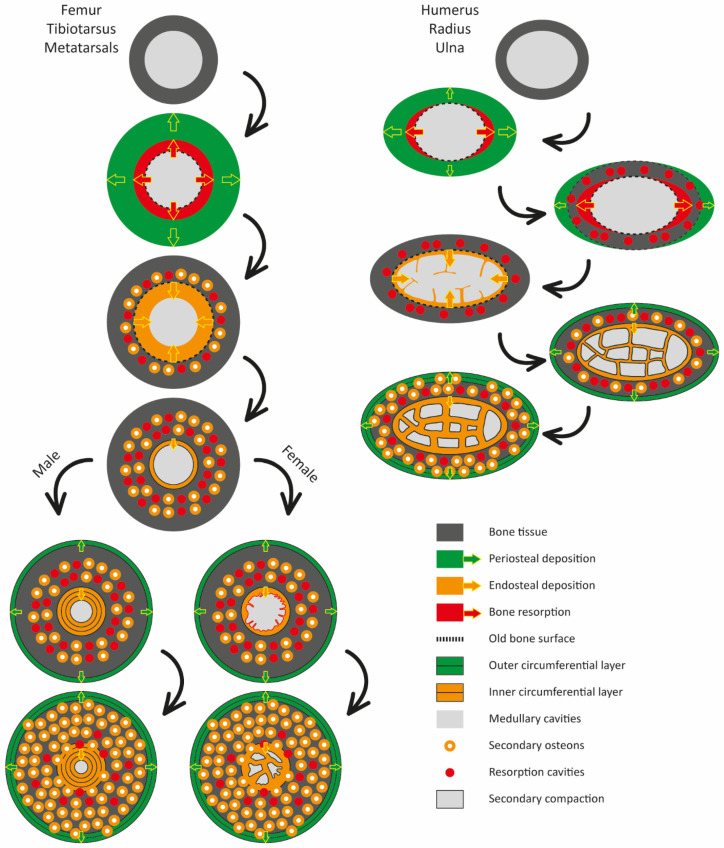
Schematic representation of the ontogenetic development of the forelimb and hindlimb skeletal elements in *Pygoscelis*. Illustration inspired by Figure 1 in Canoville et al. [11], with references taken from that work.

Analysis of the ontogenetic series of *Pygoscelis antarctica* indicates that the medullary cavity of the humerus (and probably of the entire forelimb) progressively decreases in size and becomes segmented into multiple intertrabecular spaces, independently of sex. These spaces are subsequently compacted through centripetal deposition of lamellar tissue, following a pattern comparable to the development of an ICL (Garat et al. [16], Figure 3C, Figure 4D,G,H and Figure 6). By contrast, the developmental pattern documented for the hindlimbs of both females and males (Figure 7) allows us to propose a novel aspect of sex-related bone compaction dynamics within Sphenisciformes. Unlike the condition observed in the forelimbs, the pattern expressed in the hindlimbs, particularly in females, may inhibit the formation of an ICL and instead favor the continuation of the osteosclerotic trend of the bone tissue through the development of trabecular tissue in the medullary region and its subsequent secondary compaction (Figure 7). Conversely, in the forelimbs or in skeletal elements with a flattened morphology (e.g., humerus, radius, ulna, carpometacarpus, vertebral ribs), mechanical forces associated with swimming appear to generate a dynamic tissue response, reorganizing the internal framework into a trabecular configuration and segmenting the medullary cavity into multiple intertrabecular spaces, as previously reported. Taken together, this evidence suggests that bone compaction dynamics in Sphenisciformes arise from the interaction between sex-related factors [16,24] and mechanical factors linked to locomotor activity [11]. The relative influence of these factors differs between forelimbs and hindlimbs, and between sexes, providing a coherent framework for interpreting the microstructural variability observed within the group.

## 5. Conclusions

The osteohistological comparison between *Aptenodytes patagonicus* and *Pygoscelis antarctica* reveals microstructural differences that reflect the influence of lifestyle and activity patterns on skeletal development in both species. These observations suggest that differences in the degree of bone compaction between forelimbs and hindlimbs in both extinct and extant Sphenisciformes are closely linked to locomotor strategies and adaptations to the aquatic environment.

These interpretations should be considered preliminary for the juvenile and adult stages due to limited sample representation. Future studies, including a larger number of specimens, will be necessary to validate these patterns.

In *P. antarctica*, the differential compaction of forelimbs and hindlimbs appears to be associated with the early independence of chicks, which begin marine foraging excursions between 4 and 7 weeks of age, resulting in early intensive use of the forelimbs for swimming. This activity likely provides a mechanical stimulus that accelerates bone compaction in the humerus, and probably throughout the entire forelimb, relative to the hindlimbs. By contrast, this pattern emerges at a later ontogenetic stage in *A. patagonicus*.

Moreover, in *P. antarctica*, the medullary cavity of the forelimb progressively decreases and becomes segmented during development, independently of sex. This pattern suggests that mechanical factors associated with swimming in flattened skeletal elements may promote trabecular reorganization and segmentation of the medullary cavity.

Finally, for the hindlimbs of pygoscelids, two sex-related pathways of bone compaction are proposed: in males, the medullary region continues to develop an inner circumferential layer, whereas in females, trabecular tissue develops in the medullary region and is subsequently secondarily compacted.

## Figures and Tables

**Table 1:** Ontogenetic series of *Pygoscelis antarctica*. Osteological collection of the Ornithology Section of the Vertebrate Zoology Division at Museo de La Plata (MLP-O). Histological collection of the Ornithology Section (CHO).

Collection Number	Species	Bone Element	Histological Collection Number
MLP-O 930	*Pygoscelis antarctica* (Adult)	Humerus	CHO 04-01
		Femur	CHO 04-02
		Tibiotarsus	CHO 04-03
		Vertebral rib	CHO 04-04
		Sternal rib	CHO 04-05
MLP-O 793	*Pygoscelis antarctica* (Juvenile)	Humerus	CHO 05-01
		Radius	CHO 05-02
		Ulna	CHO 05-03
		Carpometacarpus	CHO 05-04
		Femur	CHO 05-05
		Tibiotarsus	CHO 05-06
		Tarsometatarsus	CHO 05-07
		Vertebral rib	CHO 05-08
		Sternal rib	CHO 05-09
MLP-O 792	*Pygoscelis antarctica* (Chick V)	Humerus	CHO 06-01
		Radius	CHO 06-02
		Ulna	CHO 06-03
		Carpometacarpus	CHO 06-04
		Tibiotarsus	CHO 06-05
		Tarsometatarsus	CHO 06-06
		Vertebral rib	CHO 06-07
		Sternal rib	CHO 06-08
MLP-O 814	*Pygoscelis antarctica* (Chick IV)	Humerus	CHO 07-01
		Radius/Ulna	CHO 07-02
		Radius/Ulna	CHO 07-03
		Carpometacarpus	CHO 07-04
		Femur	CHO 07-05
		Tibiotarsus	CHO 07-06
		Tarsometatarsus	CHO 07-07
		Vertebral rib	CHO 07-08
		Sternal rib	CHO 07-09
MLP-O 809	*Pygoscelis antarctica* (Chick III)	Humerus	CHO 08-01
		Radius/Ulna	CHO 08-02
		Carpometacarpus	CHO 08-03
		Femur	CHO 08-04
		Tibiotarsus	CHO 08-05
		Tarsometatarsus	CHO 08-06
		Vertebral rib	CHO 08-07
		Sternal rib	CHO 08-08
MLP-O 2342	*Pygoscelis antarctica* (Chick II)	Humerus	CHO 09-01
		Radius	CHO 09-02
		Ulna	CHO 09-03
		Carpometacarpus	CHO 09-04
		Femur	CHO 09-05
		Tibiotarsus	CHO 09-06
		Tarsometatarsus	CHO 09-07
		Vertebral rib	CHO 09-08
		Sternal rib	CHO 09-09
MLP-O 14949	*Pygoscelis antarctica* (Chick I)	Femur	CHO 10-01
		Femur	CHO 10-02
		Tibiotarsus	CHO 10-03
		Vertebral rib	CHO 10-04

## Data Availability

The original contributions presented in this study are included in the Supplementary Materials. Further inquiries can be directed to the corresponding authors.

## Supplementary Materials

The following supporting information can be downloaded at https://www.mdpi.com/article/10.3390/biology15090703/s1, Figure S1: Microanatomy of histological sections of *Pygoscelis antarctica* in different bone elements analysed. From top to bottom: Adult (CHO 04 series); Juvenile (CHO 05 series); Chick V (CHO 06 series); Chick IV (CHO 07 series); Chick III (CHO 08 series); Chick II (CHO 09 series); Chick I (CHO 10 series). Cross-sectional view of the bone elements. Scale 1 cm; Figure S2: (A,B) radius/ulna of Chick III (CHO 08-02), (C,D) carpometacarpus of Chick IV (CHO 07-04) and (E,F) carpometacarpus of Chick V (CHO 06-04). (A,C,E) microanatomical features of the radius/ulna and carpometacarpus of *Pygoscelis antarctica*. (B,D,F) cortical region, under normal transmitted light (left) and under polarized light (right). (cc) calcified cartilage, (is) intertrabecular space, (kl) Kastschenko line, (po) primary osteon and (vc) vascular canal. Scale 200 μm; Figure S3: (A,B) tarsometatarsus of Chick II (CHO 09-07), (C,D) tarsometatarsus of Chick III (CHO 08-06) and (E,F) tarsometatarsus of Chick IV (CHO 07-07). (A,C,E) microanatomical features of the tarsometatarsus of *Pygoscelis antarctica*. (B,D,F) medullary region, under normal transmitted light (left) and under polarized light (right). (cc) calcified cartilage, (is) intertrabecular space, (tb) trabecular bone and (vc) vascular canal. Scale 200 μm.
