# Supplementary material for: Microstructural Variations in the Bone of Pygoscelis antarctica (Aves, Sphenisciformes) During the Postnatal Ontogeny
Source: Biology (Basel). 2026 Apr 30;15(9):703. doi: 10.3390/biology15090703 (PMC13162932; doi:10.3390/biology15090703)
Supplement: Supplementary file 1 [file biology-15-00703-s001.zip › biology-4228562-supplementary.pdf]

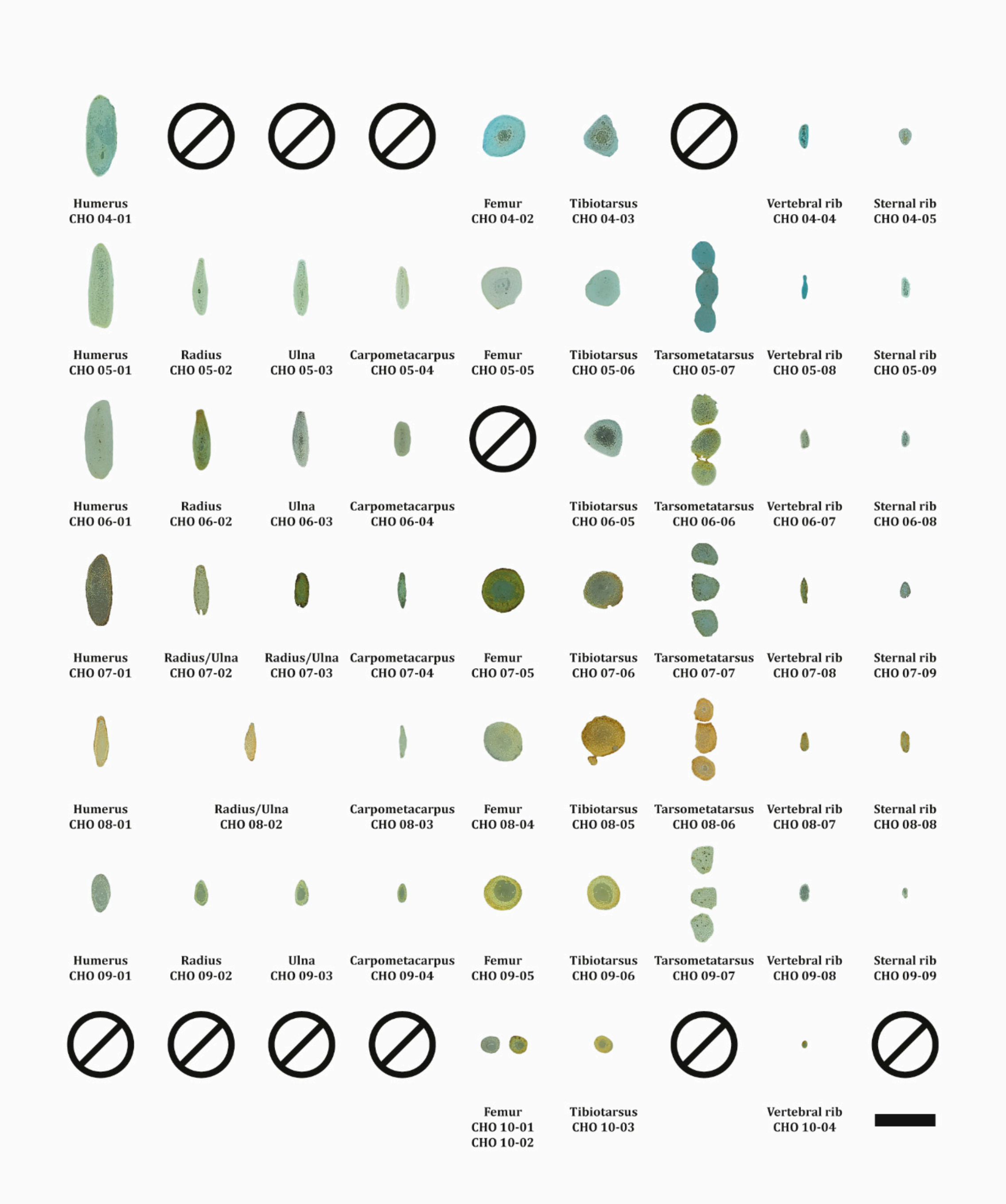

Supplementary Figure S1. Microanatomy of histological sections of *Pygoscelis antarctica* in different bone elements analysed. From top to bottom: Adult (CHO 04 series); Juvenile (CHO 05 series); Chick V (CHO 06 series); Chick IV (CHO 07 series); Chick III (CHO 08 series); Chick II (CHO 09 series); Chick I (CHO 10 series). Cross-sectional view of the bone elements. Scale 1 cm.

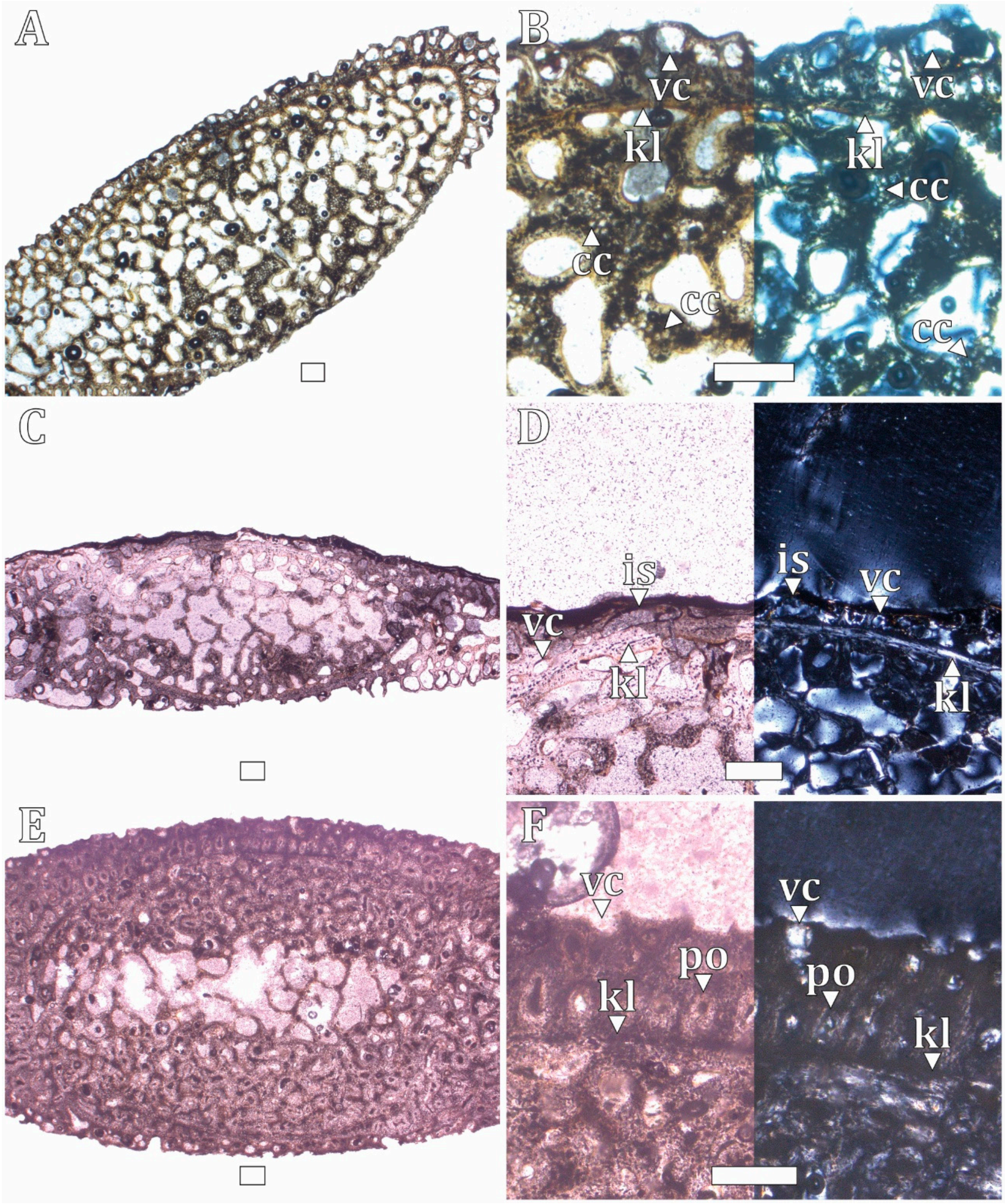

Supplementary Figure S2. (A, B) radius/ulna of Chick III (CHO 08-02), (C, D) carpometacarpus of Chick IV (CHO 07-04) and (E, F) carpometacarpus of Chick V (CHO 06-04). (A, C, E) microanatomical features of the radius/ulna and carpometacarpus of *Pygoscelis antarctica*. (B, D, F) cortical region, under normal transmitted light (left) and under polarized light (right). (cc) calcified cartilage, (is) intertrabecular space, (kl) Kastschenko line, (po) primary osteon and (vc) vascular canal. Scale 200 μm.

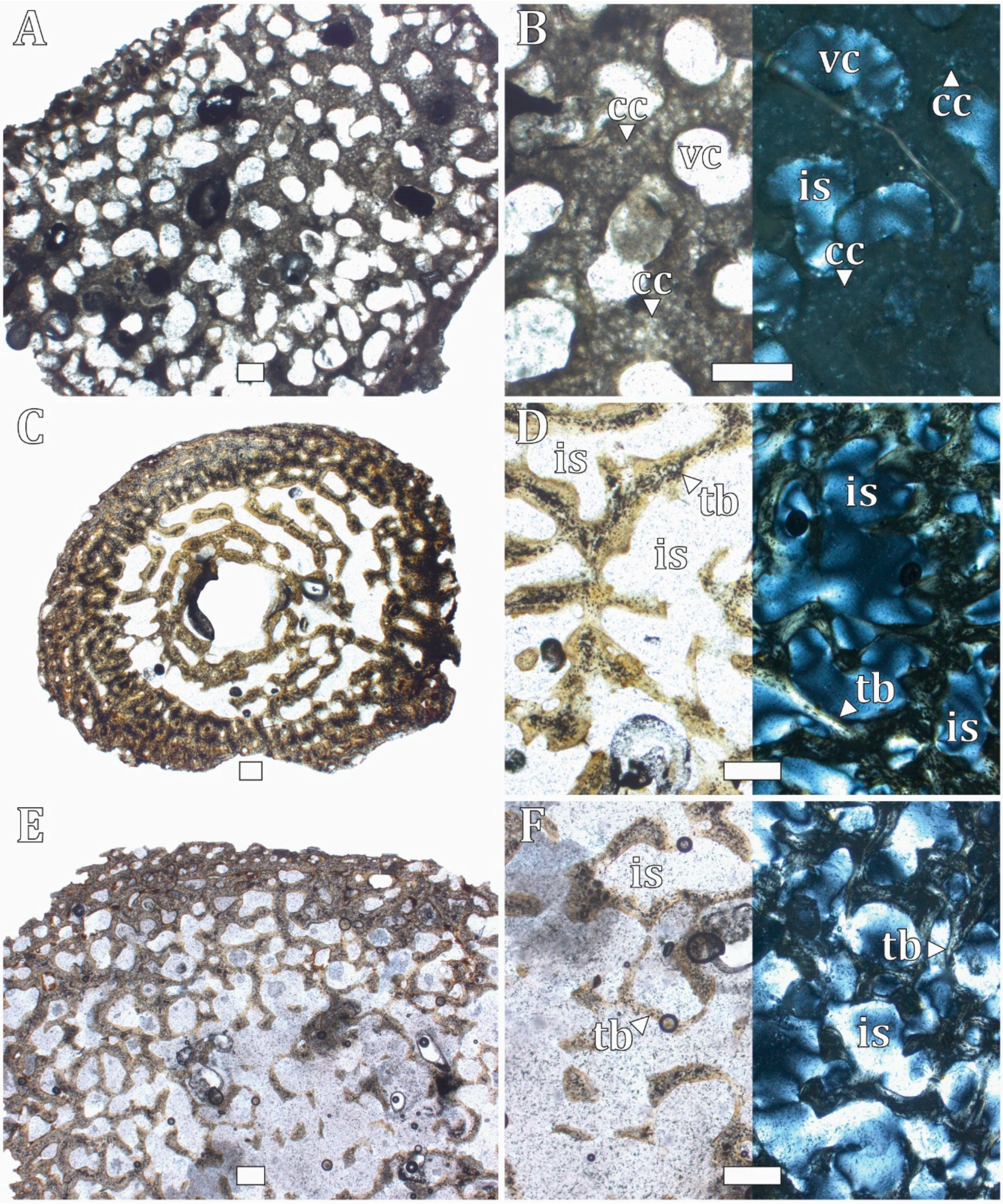

Supplementary Figure S3. (A, B) tarsometatarsus of Chick II (CHO 09-07), (C, D) tarsometatarsus of Chick III (CHO 08-06) and (E, F) tarsometatarsus of Chick IV (CHO 07-07). (A, C, E) microanatomical features of the tarsometatarsus of *Pygoscelis antarctica*. (B, D, F) medullary region, under normal transmitted light (left) and under polarized light (right). (cc) calcified cartilage, (is) intertrabecular space, (tb) trabecular bone and (vc) vascular canal. Scale 200 μm.
